# Expansion of the reducing use of sedatives (RedUSe) project to Australian nursing homes

**DOI:** 10.1186/1472-6963-14-S2-O13

**Published:** 2014-07-07

**Authors:** Juanita Westbury, Gregory Peterson, Ivan Bindoff

**Affiliations:** 1 School of Medicine, University Of Tasmania, Hobart, Tasmania, Australia

## Background

Psychotropic medications work on the brain to affect mental function and behaviour. For over 20 years, concern has been raised over the overuse of psychotropic medication, particularly antipsychotics and benzodiazepines (‘sedatives’) in nursing homes. The Reducing Use of Sedatives (RedUSe) project was developed as a multi-strategic, interdisciplinary initiative aimed to promote the quality use of sedative medication in this setting. The key strategies of RedUSe, namely audit & feedback, education and medication review, were tested in a controlled 6-month trial of 25 homes in 2008/2009. The intervention significantly reduced the rates of antipsychotic and benzodiazepine use and doubled the number of sedative dosage reductions. In addition, the rate of new sedative prescribing in intervention homes was reduced to a quarter of the rate observed in control homes. In 2013, the Australian Government awarded substantial funding to expand RedUSe to 150 nursing homes around the country. This abstract describes how the RedUse project was evaluated and enhanced before national expansion.

## Materials and methods

A thorough assessment of the barriers and enablers associated with the initial RedUSe trial was performed in line with the Theoretical Domains Framework (TDF). A qualitative methodology comprising of two focus groups with nurses and pharmacists was selected to ascertain this information. Behavioural change techniques were subsequently identified to overcome the barriers and enhance the enablers, and tested in a pilot roll out phase comprising of 27 nursing homes across three states. Feasible outcome measures were also designated.

## Results

The main barriers to the RedUSe trial were the belief that sedative medications improved resident quality of life, perceived roles of health practitioners in reviewing sedatives and poor GP engagement. The RedUSe project was enhanced by the development of a customized training program which challenged the belief that sedative use enhances resident quality of life. The training also clearly defined health practitioner roles in relation to medication review processes. The training was delivered via two facilitated interactive small group workshops which combined one didactic lecture, a case study and small group activities. An educational DVD was also produced to show at all training sessions. Academic detailing was delivered by trained detailers to inform and engage GPs.

## Conclusions

The TDF proved an effective tool to identify the key barriers and enablers to the RedUSe project, facilitating the incorporation of several novel behavioural change techniques. The success of the expanded project will be reported after full implementation and evaluation is completed.

